# Supplementary material for: Metallothionein 3 Promotes Osteoblast Differentiation in C2C12 Cells via Reduction of Oxidative Stress
Source: Int J Mol Sci. 2021 Apr 21;22(9):4312. doi: 10.3390/ijms22094312 (PMC8122383; doi:10.3390/ijms22094312)
Supplement: Supplementary file 1 [file ijms-22-04312-s001.zip › ijms-1175372-supplementary.pdf]

Supplementary Materials

to

Metallothionein 3 promotes osteoblast differentiation in C2C12 cells via reduction of oxidative stress

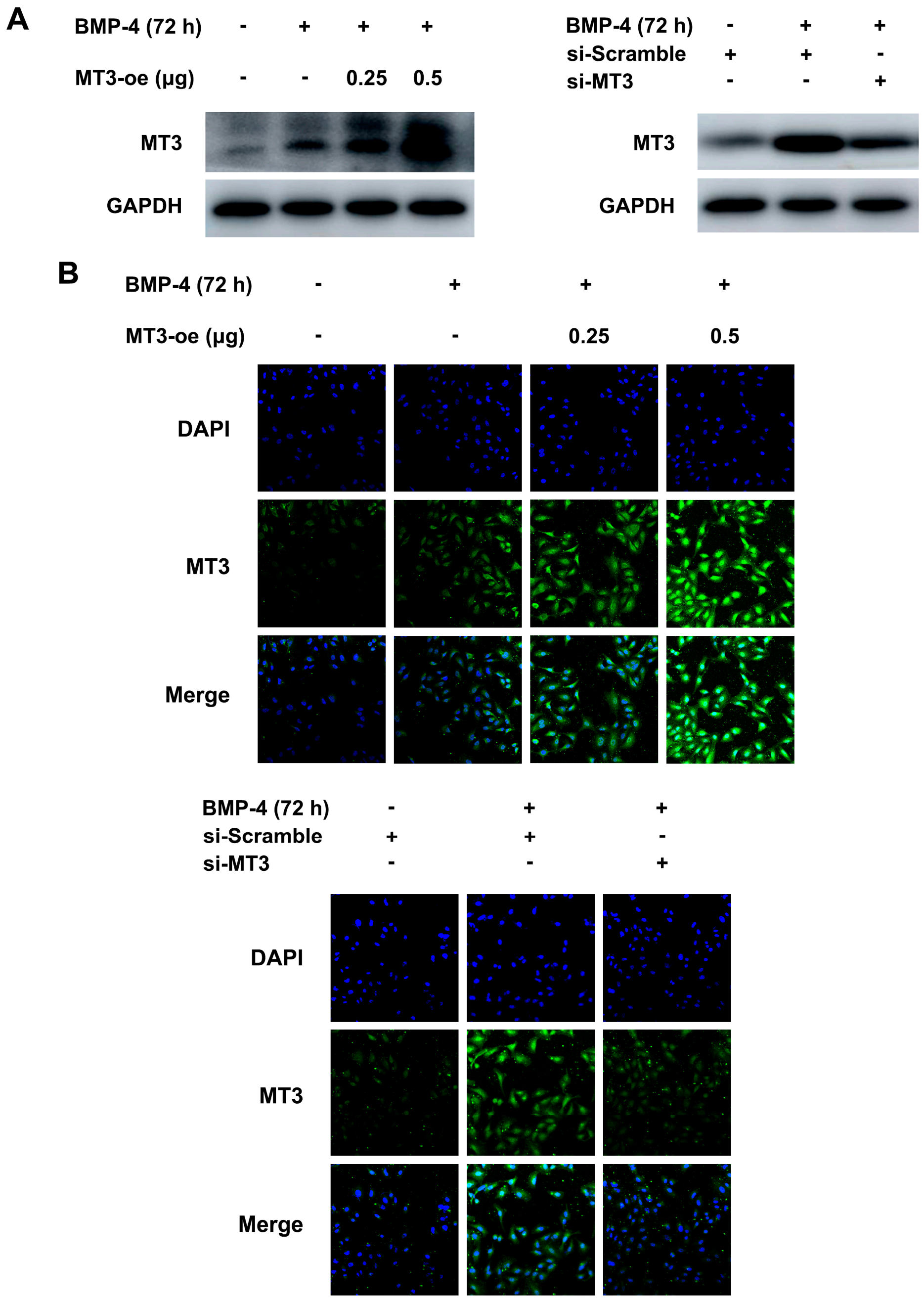

Supplementary Figure S1. Identification of the transfection efficiency of MT3-overexpressing plasmids and MT3 small interfering RNAs in C2C12 cells. (A)

Protein expression levels of MT3 and GAPDH after transfection of MT3-overexpressing plasmids or MT3 small interfering RNAs in C2C12 cells. (B) Immunofluorescence staining of MT3 after transfection of MT3-overexpressing plasmids or MT3 small interfering RNAs in C2C12 cells (magnification=200x).

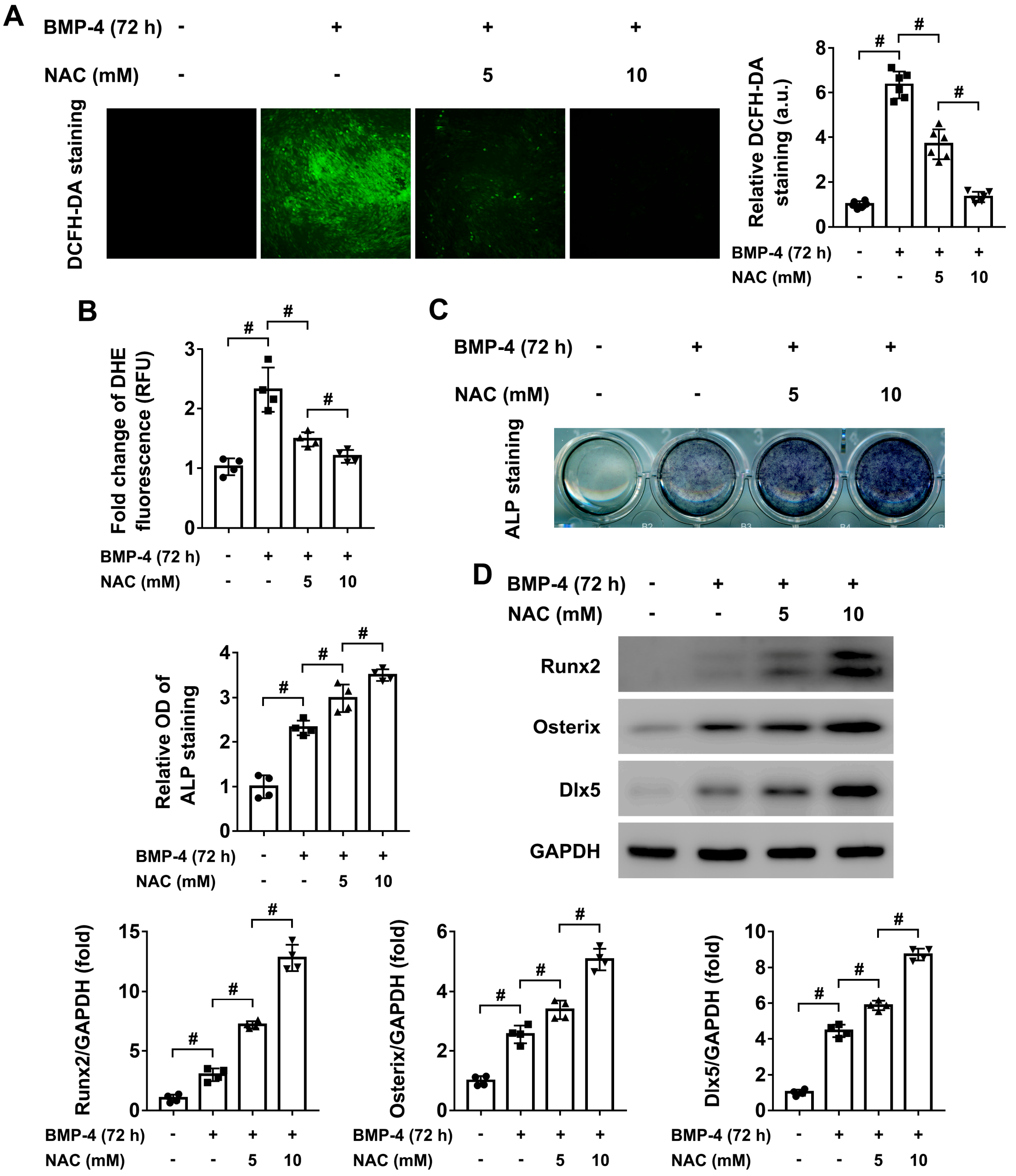

Supplementary Figure S2. NAC treatment promotes BMP4-induced osteoblast differentiation in C2C12 cells. (A) DCFH-DA staining and quantitative analysis of

ROS production in saline- or NAC-handled C2C12 cells treated with or without recombinant BMP4 for 72 hours (magnification=100x). (B) Quantitative analysis of DHE fluorescence in saline- or NAC-handled C2C12 cells treated with or without recombinant BMP4 for 72 hours. (C) ALP staining and quantitative analysis of ALP activity in saline- or NAC-handled C2C12 cells treated with or without recombinant BMP4 for 72 hours. (D) Protein expression levels and quantitative analysis of Runx2, Osterix, and Dlx5 in saline- or NAC-handled C2C12 cells treated with or without recombinant BMP4 for 72 hours. All data are presented as mean ± SEM, #P<0.05.

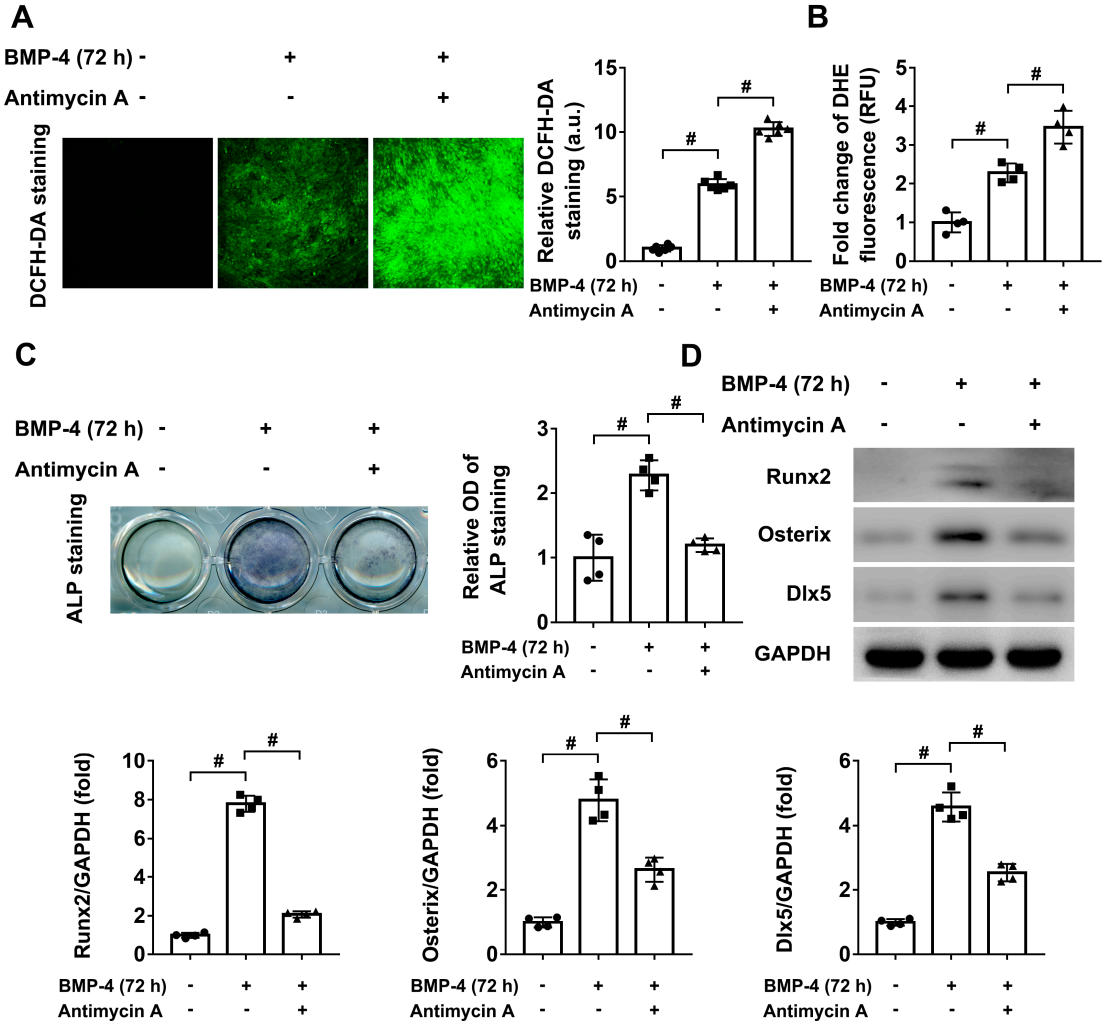

Supplementary Figure S3. Antimycin A treatment inhibits BMP4-induced osteoblast differentiation in C2C12 cells. (A) DCFH-DA staining and quantitative analysis of ROS production in saline- or antimycin A (0.5 μM)-handled C2C12 cells treated with or without recombinant BMP4 for 72 hours (magnification=100x). (B) Quantitative analysis of DHE fluorescence in saline- or antimycin A (0.5 μM)-handled C2C12 cells treated with or without recombinant BMP4 for 72 hours. (C) ALP staining and quantitative analysis of ALP activity in saline- or antimycin A (0.5 μM)-handled C2C12 cells treated with or without recombinant BMP4 for 72 hours. (D) Protein expression levels and quantitative analysis of Runx2, Osterix, and Dlx5 in saline- or antimycin A (0.5 μM)-handled C2C12 cells treated with or without recombinant BMP4 for 72 hours. All data are presented as mean ± SEM, #*P*<0.05.
